# *Chlamydia* Hijacks ARF GTPases To Coordinate Microtubule Posttranslational Modifications and Golgi Complex Positioning

**DOI:** 10.1128/mBio.02280-16

**Published:** 2017-05-02

**Authors:** Jordan Wesolowski, Mary M. Weber, Agata Nawrotek, Cheryl A. Dooley, Mike Calderon, Claudette M. St. Croix, Ted Hackstadt, Jacqueline Cherfils, Fabienne Paumet

**Affiliations:** a Department of Microbiology and Immunology, Thomas Jefferson University, Philadelphia, Pennsylvania, USA; b Host-Parasite Interactions Section, Laboratory of Bacteriology, Rocky Mountain Laboratories, National Institute of Allergy and Infectious Diseases, National Institutes of Health, Hamilton, Montana, USA; c Laboratoire de Biologie and Pharmacologie Appliquée, Centre National de la Recherche Scientifique, Ecole Normale Supérieure Paris-Saclay, Cachan, France; d Center for Biologic Imaging, University of Pittsburgh, Pittsburgh, Pennsylvania, USA; Yale University School of Medicine; University of Chicago

**Keywords:** ARF GTPase, actin, CT813, Chlamydia trachomatis, Golgi complex, inclusion protein, microtubules

## Abstract

The intracellular bacterium *Chlamydia trachomatis* develops in a parasitic compartment called the inclusion. Posttranslationally modified microtubules encase the inclusion, controlling the positioning of Golgi complex fragments around the inclusion. The molecular mechanisms by which *Chlamydia* coopts the host cytoskeleton and the Golgi complex to sustain its infectious compartment are unknown. Here, using a genetically modified *Chlamydia* strain, we discovered that both posttranslationally modified microtubules and Golgi complex positioning around the inclusion are controlled by the chlamydial inclusion protein CT813/CTL0184/InaC and host ARF GTPases. CT813 recruits ARF1 and ARF4 to the inclusion membrane, where they induce posttranslationally modified microtubules. Similarly, both ARF isoforms are required for the repositioning of Golgi complex fragments around the inclusion. We demonstrate that CT813 directly recruits ARF GTPases on the inclusion membrane and plays a pivotal role in their activation. Together, these results reveal that *Chlamydia* uses CT813 to hijack ARF GTPases to couple posttranslationally modified microtubules and Golgi complex repositioning at the inclusion.

## IMPORTANCE

*Chlamydia trachomatis* is an important cause of morbidity and a significant economic burden in the world. However, how *Chlamydia* develops its intracellular compartment, the so-called inclusion, is poorly understood. Using genetically engineered *Chlamydia* mutants, we discovered that the effector protein CT813 recruits and activates host ADP-ribosylation factor 1 (ARF1) and ARF4 to regulate microtubules. In this context, CT813 acts as a molecular platform that induces the posttranslational modification of microtubules around the inclusion. These cages are then used to reposition the Golgi complex during infection and promote the development of the inclusion. This study provides the first evidence that ARF1 and ARF4 play critical roles in controlling posttranslationally modified microtubules around the inclusion and that *Chlamydia trachomatis* hijacks this novel function of ARF to reposition the Golgi complex.

## INTRODUCTION

*Chlamydia trachomatis* is a Gram-negative bacterium that causes a range of diseases, depending on the serovar. Serovars D to K are the most common etiological agents of bacterial sexually transmitted infections, while serovars L1 to L3 cause sexually transmitted lymphogranuloma venerium, a more systemic disease (1). Serovars A to C cause trachoma, the leading cause of preventable infectious blindness (2). Despite the availability of antibiotics, *C. trachomatis* infections often go unnoticed and untreated, which can lead to pelvic inflammatory disease and infertility. Furthermore, antibiotic treatment can induce chlamydial persistence, which results in recurring infections over time (3, 4).

*Chlamydia* is an obligate intracellular pathogen that stimulates actin polymerization at the plasma membrane to induce its own uptake into a membrane-bound inclusion (5–8). The nascent inclusion is then trafficked along host microtubules to the microtubule-organizing center (MTOC), where it resides for the duration of the *Chlamydia* life cycle (9, 10). At the MTOC, *Chlamydia* establishes extensive interactions with the host Golgi complex. *C. trachomatis* fragments the Golgi complex into ministacks, which are then repositioned around the inclusion (11, 12). The importance of Golgi complex ministack formation in the pathogenesis of *Chlamydia* is highlighted by the fact that increasing the formation of ministacks via small interfering RNA (siRNA) depletion of the lateral Golgi complex tether protein Golgin-84 enhances the production of infectious progeny (12). *Chlamydia* then redirects exocytic Golgi complex-derived vesicles to the inclusion, and these vesicles are critical for inclusion development (13–16).

Around 12 h postinfection, the chlamydial inclusion is surrounded by a “cage” of microtubules (MTs) that controls the positioning of Golgi complex ministacks around the inclusion (11, 17). The depolymerization of MTs with nocodazole at the middle to late phase of the *Chlamydia* life cycle not only blocks the repositioning of the ministacks but also impairs the generation of infectious progeny (11). Interactions between Golgi complex ministacks and the inclusion are highly dynamic, since the ministacks reassemble around the inclusion following the removal of nocodazole (11). MT cages around the inclusion are enriched in posttranslationally modified alpha-tubulin, particularly acetylated and detyrosinated tubulin (11). Posttranslational modifications (PTMs) of MTs influence the recruitment of MT effectors that ultimately impact MT depolymerization and structure (18). PTM MTs have also been implicated in controlling the positioning of the Golgi complex around the inclusion (11). Importantly, the inhibition of MT detyrosination impairs the generation of infectious progeny and the repositioning of the Golgi complex around the inclusion, while enhanced PTM MTs increase *Chlamydia* infectivity (11). Thus, the presence of PTM MTs and the positioning of the Golgi complex around the inclusion appear intimately linked. However, the chlamydial protein(s) coordinating these processes remains unknown.

*Chlamydia* controls interactions between the inclusion and the host by incorporating ~60 type III secreted effector proteins called Incs into the inclusion membrane (19–21). Due to the inherent difficulty in genetically manipulating *Chlamydia*, the function of only a few inclusion proteins has been established (22). Here, using a knock out (KO) *C. trachomatis* strain, we demonstrate that the inclusion protein CT813 recruits and activates host ARF GTPases to control PTM MTs and the positioning of the Golgi complex around the inclusion. Since the function of CT813 is not limited to actin polymerization, as the InaC nomenclature suggests (**In**clusion protein for **ac**tin assembly [23]), we refer to InaC/CTL0184 as CT813. Together, our findings establish CT813 as a master cytoskeleton regulator that controls PTM MTs around chlamydial inclusions. Importantly, these results also indicate that ARF1 and ARF4 play significant roles in the regulation of cytoskeleton dynamics.

## RESULTS

### CT813 recruits host GTPases ARF1 and ARF4 to the inclusion membrane by direct protein-protein interactions.

**FIG 1:**
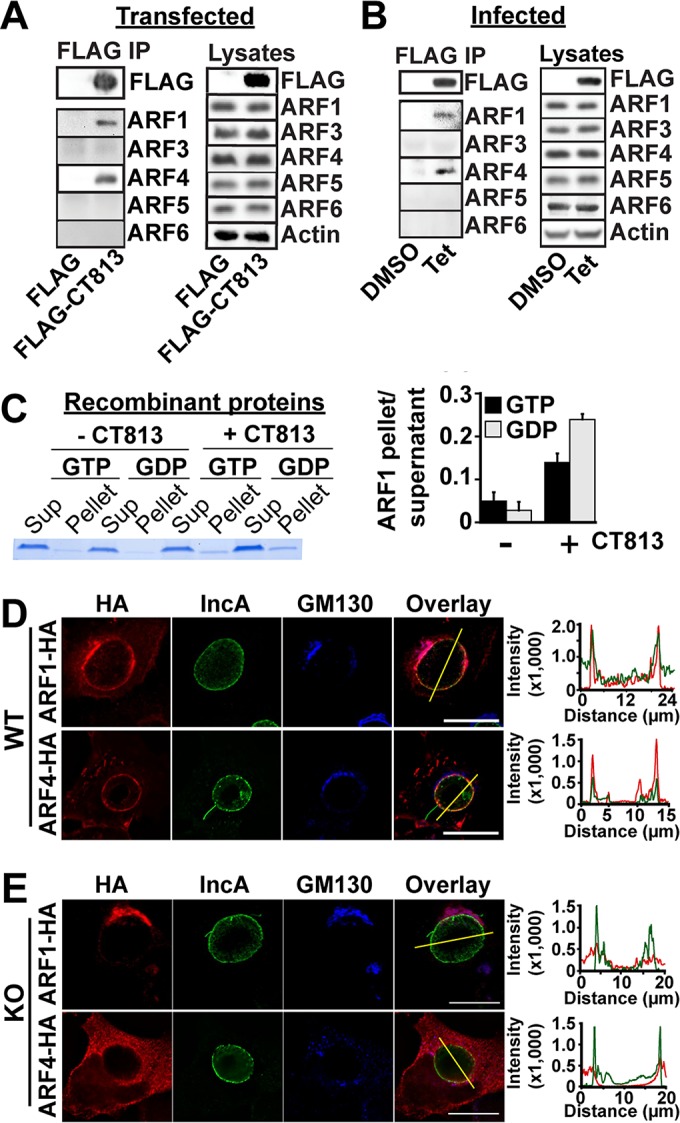
CT813 recruits host ARF GTPases to the inclusion by direct protein-protein interactions. (A) Anti-FLAG immunoprecipitation of FLAG-CT813 from transfected cells. (B) Immunoprecipitation of CT813-FLAG from cells infected with CT813-FLAG-overexpressing *Chlamydia* cells in the presence of dimethyl sulfoxide (DMSO) or Tet for 24 h. Lysates for the experiments shown in panels A and B were processed as controls. Actin was used as a loading control. (C, left) Δ17ARF1-GDP or -GTP was cosedimented with CT813-containing liposomes. Samples were then resolved by SDS-PAGE, and proteins were revealed by Coomassie staining. Sup, supernatant. (Right) Average ratio of ARF1 in the pellet versus ARF1 in the supernatant from three independent experiments ± standard deviations. (D) HeLa cells expressing ARF1-HA or ARF4-HA were infected with WT *Chlamydia* for 18 h. Cells were labeled with anti-HA (red), anti-GM130 (blue), and anti-IncA (green) antibodies. Bar, 10 μm. The line intensity scans indicate the coincidence of HA with IncA staining on WT inclusions. ARF-HA constructs are denoted as red lines, and IncA constructs are shown as green lines.

Previous work showed that ectopically expressed CT813 coimmunoprecipitates with ARF1 and that green fluorescent protein (GFP)-tagged ARF GTPases are recruited around the inclusion in a CT813-dependent manner during infection (23). We confirmed these observations using FLAG-tagged CT813 overexpressed in noninfected cells; only ARF1 and ARF4 were found to interact with CT813 in this experimental setting, suggesting a more restricted specificity (Fig. 1A, left). Furthermore, these data indicated that the interaction between CT813 and ARF occurs independently of additional chlamydial proteins, since these cells were not infected.

We validated that CT813 interacts with ARF GTPases during infection using a genetically modified strain of *C. trachomatis* overexpressing CT813-FLAG. *C. trachomatis* was stably transformed with a vector encoding both (i) CT813-FLAG under control of the tetracycline promoter and (ii) GFP driven by a constitutive *Neisseria meningitidis* promoter (see Fig. S1A in the supplemental material). The addition of anhydrotetracycline (Tet) induced CT813-FLAG expression (Fig. S1B). CT813-FLAG was then immunoprecipitated from infected cells, and the bound ARF isoforms were analyzed by Western blotting. Only ARF1 and ARF4 coimmunoprecipitated with CT813 in CT813-FLAG-tagged *Chlamydia*-infected cells (Fig. 1B), demonstrating that the interaction occurs during infection and confirming its specificity.

10.1128/mBio.02280-16.1 FIG S1  Generation of CT813 *Chlamydia* clones. (A) Map of vectors used to generate CT813-FLAG-overexpressing *C. trachomatis*. CT813-FLAG is under tight control by the Tet promoter. These clones also constitutively express GFP. (B) Confirmation of CT813-FLAG overexpression. (Left) Lysates from HeLa cells infected with CT813-FLAG *C. trachomatis* cells were prepared at 24 h postinfection (hpi) and analyzed by Western blotting. CT813-FLAG expression was induced with 10 ng/ml Tet or DMSO as a control 8 hpi. (Right) Infected cells were fixed 24 hpi and labeled with anti-FLAG antibody (red), and DNA was labeled with Hoechst. Asterisks indicate inclusions. Constitutive expression of GFP is shown in green. (C) Experimental design of TargeTron-based inactivation of CT813. (D) Confirmation of CT813 inactivation. (Left) Successful incorporation of the beta-lactamase resistance cassette (higher band) was determined by PCR. (Center) Western blot analysis of CT813 expression shows complete knockout of CT813 expression. (Right) CT813 was not detected on the inclusion membrane by immunofluorescence microscopy. Asterisks denote inclusions. Scale bar = 20 μm. (E) Analysis of inclusion-forming units for wild-type (WT) and CT813 KO *C. trachomatis* at 48 hpi. Data were normalized to results with WT (set as 100%). **, *P* ≤ 0.01. Download FIG S1, TIF file, 1.2 MB. Copyright © 2017 Wesolowski et al. 2017 Wesolowski et al. This content is distributed under the terms of the Creative Commons Attribution 4.0 International license.

Next, we tested whether CT813 and ARF GTPases directly interact in a liposome recruitment assay with purified recombinant CT813 and ARF1 proteins. In this assay, we analyzed whether a truncated version of ARF that cannot bind to membranes on its own (Δ17ARF1) was recruited to liposomes in which CT813 had been reconstituted. Both Δ17ARF1-GDP and Δ17ARF1-GTP were recruited to CT813-containing liposomes (Fig. 1C), indicating that CT813 interacts directly with ARF and that this interaction occurs irrespective of the nature of the bound nucleotide and independently of other cellular or bacterial factors.

Finally, we determined the localization of CT813-recruited ARF1 and ARF4 during infection by using cells transfected with low levels of hemagglutinin (HA)-tagged ARF1 and ARF4. To discriminate between ARF1 and ARF4 bound to Golgi complex ministacks and those present on the inclusion membrane, we analyzed regions of the inclusion membrane where Golgi complex ministacks were absent. As shown in Fig. 1D, both ARF1 and ARF4 colocalized with the inclusion membrane marker IncA, indicating that they are recruited to the inclusion membrane. Consistent with the immunoprecipitation data, ARF3, ARF5, and ARF6 were not recruited to wild-type (WT) inclusions (Fig. S2). While GFP-tagged ARFs 1 to 5 have been described on the inclusion (23), large tags like GFP can disrupt ARF function and are likely the cause of the discrepancy (24). Using a *Chlamydia* strain in which CT813 expression was knocked out via group II intron-based insertional inactivation (Fig. S1C and D), we observed that ARF1 and ARF4 did not localize to the inclusion, demonstrating that CT813 is required for ARF recruitment to the inclusion (Fig. 1E). Based on these findings, we conclude that CT813 specifically interacts with and recruits ARF1 and ARF4 to the inclusion membrane during infection.

10.1128/mBio.02280-16.2 FIG S2  ARFs 3, 5, and 6 are not recruited to WT *Chlamydia* inclusions. Cells were transfected with HA-tagged ARF constructs prior to infection with WT *Chlamydia*. Cells were fixed 18 h postinfection (hpi) and stained with anti-HA (red) antibody. The inclusion membrane was labeled with anti-IncA (green) antibody, and DNA was stained with Hoechst (gray). Scale bar = 10 µm. The line intensity scans indicate that HA is not coincident with IncA on WT inclusions. ARF-HA constructs are denoted as red lines, and IncA is shown as green lines. Download FIG S2, TIF file, 0.7 MB. Copyright © 2017 Wesolowski et al. 2017 Wesolowski et al. This content is distributed under the terms of the Creative Commons Attribution 4.0 International license.

### CT813, ARF1, and ARF4 control Golgi complex positioning during infection.

**FIG 2:**
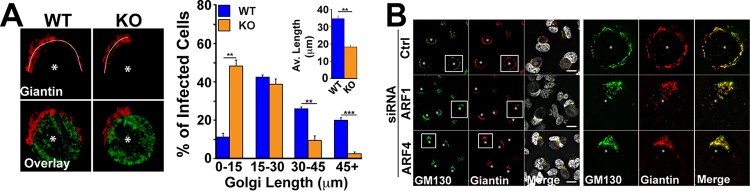
CT813, ARF1, and ARF4 control Golgi complex positioning during infection. (A, left) Cells infected with the indicated *Chlamydia* strains were fixed 24 h postinfection and stained with anti-giantin (red) and anti-LPS (green) antibodies. The images correspond to maximum projections. Asterisks denote inclusions. Bar, 5 μm. The white line indicates how the Golgi complex was measured. (Right) The percentages of infected cells containing a Golgi complex of the indicated size from five independent experiments ± standard deviations (SD). The inset denotes average Golgi complex sizes from five independent experiments ± SD. A minimum of 100 cells per condition was counted for each experiment. **, *P* ≤ 0.01; ***, *P* ≤ 0.001. (B) HeLa cells were treated with the indicated siRNAs for 48 h prior to infection with WT *Chlamydia*. Cells were fixed 24 h postinfection and labeled with anti-GM130 (green) and anti-giantin (red) antibodies to label the Golgi complex. Asterisks indicate inclusions. Bar, 20 μm.

During infection, *C. trachomatis* fragments the Golgi complex into ministacks that subsequently surround the inclusion (11). This reorganization of the Golgi complex is critical for inclusion development (12). The CT813 KO *Chlamydia* strain displayed smaller inclusions (Fig. S3) and produced fewer infectious progeny (Fig. S1E). Since ARF GTPases are major regulators of Golgi complex structure in mammalian cells (25), we hypothesized that CT813 may divert ARF1 and ARF4 to manipulate Golgi complex membranes to promote inclusion development and replication. To address this hypothesis, we first determined the role of CT813 in Golgi complex positioning during infection by using WT and CT813 KO *Chlamydia*-infected cells. In WT *Chlamydia*-infected cells, Golgi complex fragments spread to >45 μm in length around the inclusion (Fig. 2A). In contrast, the Golgi complex remained compact in the CT813 KO *Chlamydia*-infected cells, with 50% to 60% of the cells exhibiting a Golgi complex of ≤15 μm, compared to only 10% in WT-infected cells (Fig. 2B). These observations indicate that CT813 plays a critical role in Golgi complex positioning during *Chlamydia* infection.

10.1128/mBio.02280-16.3 FIG S3  CT813 controls inclusion growth. (Left) HeLa cells were infected with WT or CT813 KO *C. trachomatis* for 24 h prior to fixation and labeling with anti-LPS antibody (green). Scale bar = 20 μm. (Right) The normalized average inclusion area, from four independent experiments ± standard deviations. A minimum of 100 cells per condition was measured for each experiment. *, *P* ≤ 0.05. Download FIG S3, TIF file, 0.3 MB. Copyright © 2017 Wesolowski et al. 2017 Wesolowski et al. This content is distributed under the terms of the Creative Commons Attribution 4.0 International license.

Next, we used siRNA to test whether ARF1 and ARF4 are also required for Golgi complex positioning during WT *Chlamydia* infection (Fig. S4B). In infected control cells, both GM130 (*cis*) and Giantin (medial) Golgi complex markers labeled the Golgi complex ministacks surrounding the inclusion (Fig. 2B, Ctrl siRNA). This observation is consistent with the typical distribution of the Golgi complex during *Chlamydia* infection (12). In contrast, the Golgi complex remained compact in either ARF1 or ARF4 siRNA-treated cells (Fig. 2B, ARF1 siRNA and ARF4 siRNA), demonstrating that both ARF isoforms are involved in Golgi complex positioning during infection and that their functions are not redundant. Note that Golgi complex morphology in noninfected cells is not affected by ARF1- or ARF4-siRNA treatment, suggesting that the effect observed during infection is not due to a global Golgi complex defect (data not shown) (26). Altogether, these data suggest that CT813 uses ARF1 and ARF4 to control Golgi complex positioning during *Chlamydia* infection.

10.1128/mBio.02280-16.4 FIG S4  Alpha-tubulin cage formation is independent from CT813, ARF1, and ARF4. (A) HeLa cells were infected with the indicated *Chlamydia* strains for 24 h. Cells were fixed and labeled with alpha-tubulin antibody (green). DNA was stained with Hoechst. Scale bar = 20 μm. (B) HeLa cells were treated with the indicated siRNAs for 48 h prior to lysis and analysis by RT-PCR (left) or Western blotting (right). PPIA (RT-PCR) and HSP70 (WB) were used as loading controls. (C) HeLa cells were treated with the indicated siRNAs for 48 h prior to infection with WT *Chlamydia*. Cells were fixed and labeled with alpha-tubulin antibody (green). DNA was stained with Hoechst. Scale bar = 20 μm. Download FIG S4, TIF file, 2.5 MB. Copyright © 2017 Wesolowski et al. 2017 Wesolowski et al. This content is distributed under the terms of the Creative Commons Attribution 4.0 International license.

### CT813 and ARF GTPases cooperate to stabilize microtubules around the inclusion.

**FIG 3:**
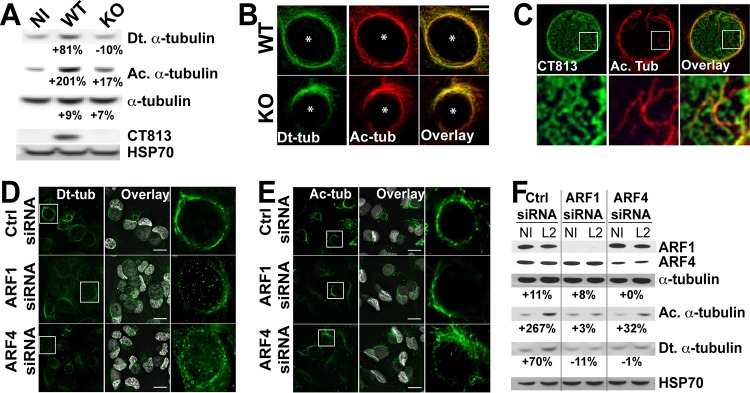
CT813, ARF1, and ARF4 cooperate to stabilize MTs around the inclusion. (A) Cells infected with the indicated *Chlamydia* strains were lysed 24 h postinfection (hpi), and samples were analyzed by Western blotting. HSP70 served as a loading control. Numbers indicate the percent change in the indicated tubulin with respect to HSP70 and compared to the noninfected control. Results are representative of three independent experiments. (B) WT and CT813 KO *Chlamydia*-infected cells were fixed 24 hpi and labeled with anti-detyrosinated tubulin (Dt-tub; green) and anti-acetylated tubulin (Ac-tub; red) antibodies. Asterisks denote inclusions. Bar, 5 μm. (C) High-resolution microscopy images of WT *Chlamydia*-infected cells fixed 24 h pi and labeled with anti-acetylated tubulin (Ac. Tub; red) and anti-CT813 (green) antibodies. (D and E) HeLa cells were treated with the indicated siRNAs for 48 h prior to infection with WT *Chlamydia*. Cells were fixed 24 hpi and labeled with anti-detyrosinated tubulin (Dt-tub; green) (D) or anti-acetylated tubulin (Ac-tub; green) (E) antibodies. DNA was stained with Hoechst. Bar, 20 μm. (F) siRNA-treated infected cells were lysed 24 hpi, and samples were analyzed by Western blotting. HSP70 served as a loading control. Numbers indicate the percent change in the indicated tubulin with respect to HSP70 in comparison with noninfected cells for each condition. Results are representative of three independent experiments.

*Chlamydia* remodels PTM MTs to recruit Golgi complex ministacks to the inclusion (11). Our observation that CT813, ARF1, and ARF4 are key players in Golgi complex positioning thus raised the possibility that they are involved in the manipulation of MTs during *Chlamydia* infection. First, we assessed whether the structure of alpha-tubulin cages was dependent on CT813 or ARF1 and ARF4. The total amount of alpha-tubulin, as well as the structure of alpha-tubulin cages, remained unaffected by the depletion of CT813 (Fig. 3A; Fig. S4A). Similarly, siRNA-mediated depletion of ARF1 or ARF4 had no significant impact on alpha-tubulin cages or protein levels (Fig. 3F; Fig. S4B), suggesting that CT813, ARF1, and ARF4 do not control the formation of MT cages.

Next, we analyzed the amount of PTM tubulin. As previously observed (11), the amount of both detyrosinated and acetylated alpha-tubulin increased by 81% and 201%, respectively, during WT *Chlamydia* infection compared to the noninfected control (Fig. 3A, NI and WT) (11). Remarkably, the amount of PTM MTs in CT813 KO *Chlamydia*-infected cells was significantly reduced compared to that in WT-infected controls (Fig. 3A, KO), demonstrating that CT813 is involved in the generation of PTM MTs during infection. Next, we assessed the organization of PTM MTs during infection. Using immunofluorescence microscopy, we detected a significant enrichment of detyrosinated and acetylated tubulin cages around WT inclusions (Fig. 3B, WT). In contrast, detyrosinated and acetylated tubulin cages were disorganized in CT813 KO *Chlamydia*-infected cells, leading to the incomplete enclosure of the PTM MT cages around the inclusion and disheveled structures (Fig. 3B, KO), which correlated with the decrease in the amount of PTM MTs. While the PTM MTs around CT813 KO inclusions were impaired, PTM MTs remained associated with the Golgi complex (Fig. S5), suggesting that there is not a global defect in PTM MTs. Interestingly, using high-resolution microscopy we observed that CT813 was expressed on the inclusion surface in discrete microdomains (Fig. 3C) and that acetylated tubulin appeared to form direct contacts with the inclusion via these CT813 microdomains.

10.1128/mBio.02280-16.5 FIG S5  PTM MTs remain associated with the Golgi complex in CT813 KO-infected cells. HeLa cells were infected with the indicated strains for 24 h, followed by fixation and staining with anti-detyrosinated tubulin (Dt-tub; green) and anti-GM130 (red; Golgi complex) antibodies. Asterisks denote inclusions. Scale bar = 5 μm. Download FIG S5, TIF file, 0.4 MB. Copyright © 2017 Wesolowski et al. 2017 Wesolowski et al. This content is distributed under the terms of the Creative Commons Attribution 4.0 International license.

Finally, we investigated the contribution of ARF1 and ARF4 to the formation of PTM MT cages during infection. As shown in Fig. 3D and E, both types of PTM MT cages were significantly impaired in ARF1 or ARF4 siRNA-treated cells infected with WT *Chlamydia*, leading to incomplete and disheveled PTM MT structures around the inclusion. Additionally, the amount of acetylated and detyrosinated tubulin failed to increase in ARF-depleted cells infected with WT *Chlamydia* (Fig. 3F, compare data for anti-acetylated alpha-tubulin [Ac-tub] and anti-detyrosinated alpha-tubulin [Dt-tub] in Ctrl versus ARF1 and ARF4 siRNA-treated cells). These observations demonstrated that both ARF1 and ARF4 play critical roles in the induction of PTM MT cages around the inclusion, which phenocopies the role of CT813.

### CT813-dependent PTM MT cage formation is required for Golgi complex repositioning.

A role for CT813 in the formation of actin cytoskeletal cages around the inclusion was recently identified in a chemically mutagenized strain (23). We confirmed this observation in the CT813 KO strain (Fig. S6A, WT and KO). Since the cytoskeleton is intimately connected and the CT813 KO strain loses both actin and PTM MT cages, we investigated the roles of actin and PTM MT cages in Golgi complex repositioning by using the CT813-FLAG-overexpressing strain. Similar to the CT813 KO strain, the overexpression of CT813-FLAG impaired Golgi complex positioning around the inclusion by ~50% (Fig. S6B). Likewise, the induction of PTM MTs (Fig. S6C) and the formation of PTM MT cages (Fig. S6D) were impaired, suggesting that the overexpression of CT813-FLAG functions in a dominant-negative manner. Surprisingly, CT813-FLAG-overexpressing inclusions were still surrounded by actin cages (Fig. S6A, DMSO and Tet). These data indicate that it is likely the loss of PTM MT cages and not actin cages in the CT813 KO strain that is responsible for the Golgi complex positioning defect; this further supports a central role for CT813-dependent induction PTM MTs and PTM MT cage formation in Golgi complex positioning around the inclusion.

10.1128/mBio.02280-16.6 FIG S6  CT813-dependent PTM MT cage formation is required for proper Golgi complex positioning. (A) Cells were infected with the indicated strains for 32 h. Cells were fixed and stained with phalloidin (green) to label actin. Scale bar = 20 µm. Asterisks denote inclusions. (B, left) HeLa cells were infected with CT813-FLAG-overexpressing *Chlamydia* cells for 24 h. CT813-FLAG was induced 8 h postinfection (hpi) with 10 ng/ml anhydrotetracycline (Tet). Dimethyl sulfoxide (DMSO) served as a control. Cells were fixed and stained with anti-giantin (red) to label the Golgi complex. This strain constitutively expresses GFP (green). The images correspond to maximum projections. The white line indicates how the Golgi complex was measured. Asterisks denote inclusions. (Right) Average lengths of the Golgi complex from five independent experiments ± the standard deviation. **, *P* ≤ 0.01. (C) Cells were infected and induced as described for panel B, lysed, and samples were analyzed by Western blotting using the indicated antibodies. NI, not infected. (D) Cells were infected and induced as described for the experiment shown in panel B, fixed, and stained with anti-detyrosinated tubulin (Dt-tub; green) and anti-acetylated tubulin (Ac-tub; red) antibodies. Download FIG S6, TIF file, 2.4 MB. Copyright © 2017 Wesolowski et al. 2017 Wesolowski et al. This content is distributed under the terms of the Creative Commons Attribution 4.0 International license.

### *Chlamydia* activates ARF in a CT813-dependent manner.

**FIG 4:**
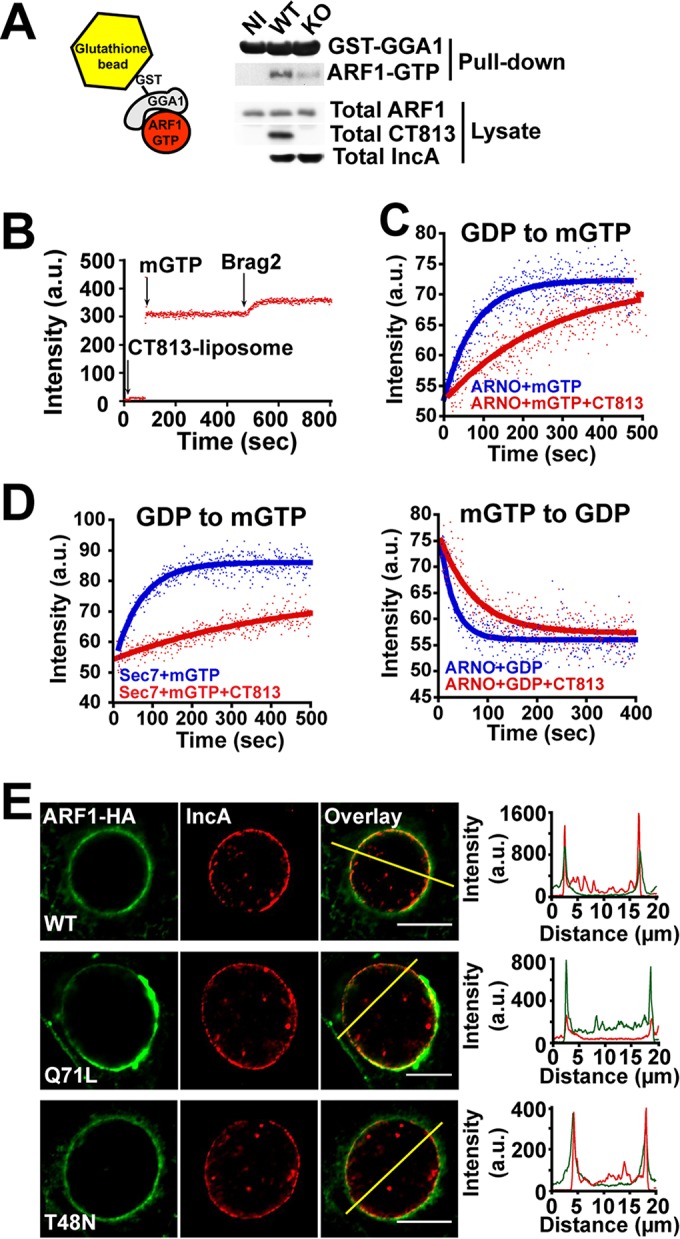
Chlamydia activates ARF in a CT813-dependent manner. (A) Cells were infected with the indicated *Chlamydia* strains at a multiplicity of infection of 5 for 24 h. The levels of ARF-GTP in the lysates were assessed using the glutathione *S*-transferase (GST)-tagged ARF effector GGA1 as bait. The absence of ARF-GTP in the noninfected control was likely due to the low sensitivity of the ARF1 antibody. IncA, another inclusion protein, was used as an infection control. GST-GGA1 was used as a loading control. (B) CT813 and myrisotylated ARF1 were incorporated into liposomes. The addition of mGTP to the reaction mixture resulted in a nonspecific increase in fluorescence. Subsequent increases in fluorescence, indicating the exchange of GDP for mGTP on ARF1, were not observed until the GEF Brag2 was added to the reaction mixture. (C and D) Myristoylated ARF1 loaded with the indicated nucleotides was reconstituted into liposomes with or without CT813. Nucleotide exchange in the forward (GDP-to-GTP) and reverse (GTP-to-GDP) direction using the GEF ARNO (C) or an isolated Sec7 domain (D) was conducted as described in Materials and Methods. (E, left) HeLa cells were infected with WT *Chlamydia* and transfected with ARF1 WT-HA, ARF1 Q71L-HA, or ARF1 T48N-HA. Cells were fixed 24 h postinfection and labeled with anti-HA (green) and anti-IncA (red) antibodies. Bar, 20 μm. (Right) Intensity line scans, indicating overlap of HA and IncA signals.

ARF GTPases cycle between GDP- and GTP-bound states. Since CT813 recruits ARF1 to the inclusion membrane (Fig. 1) and the nucleotide-bound state of ARF controls its activity (27), we determined the activation state of ARF during *Chlamydia* infection. GTP-bound ARF was isolated from noninfected and *Chlamydia*-infected cells by using the ARF effector GGA1 as bait (Fig. 4A, left). GGA1 specifically binds ARF1-GTP and is commonly used to assess ARF activation (28). We observed increased levels of ARF1-GTP upon infection with WT *Chlamydia* and significantly reduced levels of ARF-GTP in CT813 KO-infected lysates (Fig. 4A, KO versus WT). These results indicate that *Chlamydia* induces ARF activation in a CT813-dependent manner.

The increase of ARF-GTP levels could result from CT813 functioning as a guanine nucleotide exchange factor (GEF) to activate ARF. To investigate this possibility, we carried out a fluorescence-based *in vitro* kinetics assay in which GDP/GTP exchange of myristoylated ARF1-GDP was measured. Recombinant CT813 and myrisotylated ARF1 were incorporated into liposomes. The addition of 2′,3′-*O*-(*N*-methyl-anthraniloyl)-guanosine-5′-triphosphate (mantGTP, or mGTP) to the reaction mixture resulted in a nonspecific increase in fluorescence. Subsequent increases in fluorescence, indicating the exchange of GDP for mGTP on ARF1, were not observed until the GEF Brag2 was added to the reaction mixture, indicating that CT813 does not function as a GEF on its own (Fig. 4B). Alternatively, CT813 could stabilize ARF-GTP by binding as an effector. To test this possibility, we took advantage of the fact that all GEFs exchange nucleotides in both the forward (GDP-to-GTP) and reverse (GTP-to-GDP) directions (29). If CT813 functions as an effector, then it would be predicted to increase forward exchange by displacing the equilibrium toward ARF-GTP and to slow reverse exchange by competing with the GEF. Surprisingly, we observed that CT813 inhibited nucleotide exchange by the ARF GEF ARNO both in the forward direction by ~4-fold (Fig. 4C, upper) and in the reverse direction by ~2-fold (Fig. 4C, lower). Inhibition of nucleotide exchange was also observed with another cellular ARF GEF, Brag2 (Fig. S7), and with the isolated Sec7 domain of ARNO (~7-fold) (Fig. 4D). These observations indicate that CT813 is not a classical effector and that its site of interaction on ARF1 overlaps with the binding site of cellular ARF GEFs.

10.1128/mBio.02280-16.7 FIG S7  CT813 inhibits Brag2-mediated nucleotide exchange. Similar to the GEF ARNO (Fig. 4C), CT813 inhibits GDP-to-mGTP (left) and mGTP-to-GDP (right) exchange mediated by the GEF Brag2. Download FIG S7, TIF file, 0.1 MB. Copyright © 2017 Wesolowski et al. 2017 Wesolowski et al. This content is distributed under the terms of the Creative Commons Attribution 4.0 International license.

To determine whether *Chlamydia* exploits this nucleotide-independent interaction, we analyzed the localization of overexpressed HA-tagged GTP-locked ARF1 (Q71L) or GDP-locked ARF1 (T48N) (30) in WT *Chlamydia*-infected cells. As shown in Fig. 4E, both ARF1 Q71L (GTP) and ARF1 T48N (GDP) were recruited to the inclusion membrane, indicating that CT813 binds to both forms and recruits them to the inclusion.

### ARF activation controls Golgi complex positioning around the inclusion and the organization of stable MTs.

**FIG 5:**
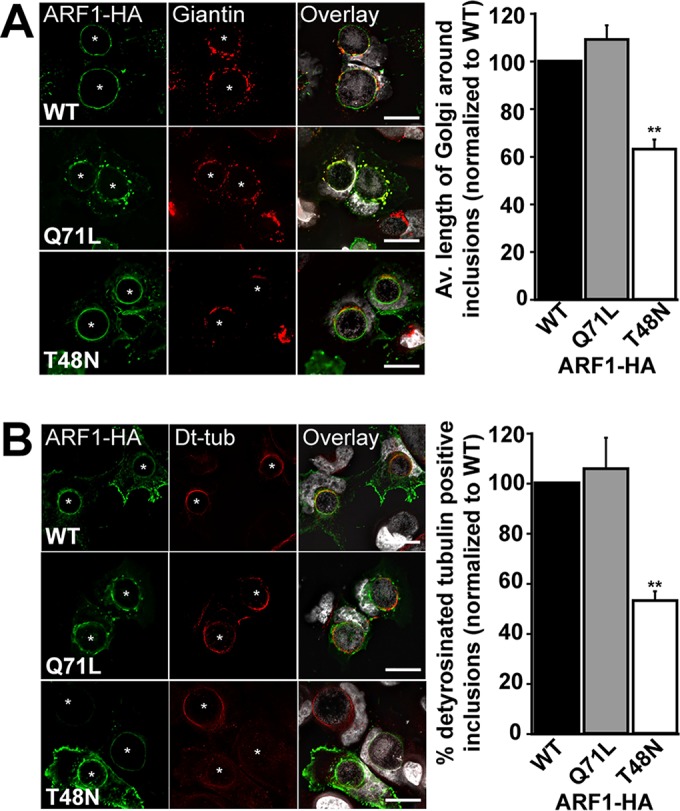
ARF activation controls Golgi complex recruitment through the formation of stable MTs. HeLa cells were infected with WT *Chlamydia* and transfected with WT, Q71L, or T48N ARF1-HA. (A) Cells were fixed 24 h postinfection (hpi) and labeled with anti-HA (green) and anti-giantin (red) antibodies. DNA was stained with Hoechst stain. Bar, 20 μm. The graph represents Golgi complex lengths in infected cells, normalized to that of ARF1 WT-HA-transfected cells from three independent experiments, ± standard deviations (SD). A minimum of 100 cells per condition was measured for each experiment. (B) Cells were fixed 24 hpi and labeled with anti-HA (green) and anti-detyrosinated tubulin (Dt-tub; red) antibodies. DNA was stained with Hoechst. Bar, 20 μm. The graph represents the percentage of cells containing Dt-tub cages, normalized to that of ARF1 WT-HA-transfected cells from three independent experiments ± SD. A minimum of 100 cells per condition was measured for each experiment. Asterisks denote inclusions. **, *P* ≤ 0.01.

Given the role of ARF GTPases in controlling Golgi complex and PTM MT organization around the inclusion and given the fact that CT813 recruits both GDP- and GTP-bound ARF1 to the inclusion membrane (Fig. 4E), we investigated the respective roles of each nucleotide-bound form during infection in cells expressing low levels of HA-tagged ARF1 WT, ARF1 Q71L (GTP locked), or ARF1 T48N (GDP locked) in WT *Chlamydia*-infected cells. First, we analyzed their impact on the repositioning of Golgi complex ministacks. The overexpression of ARF-GTP (Q71L) and WT ARF1 displayed normal Golgi complex positioning around the inclusion (Fig. 5A, WT and Q71L). In contrast, the overexpression of the GDP-locked (T48N) ARF1 inhibited Golgi complex spreading around the inclusion by ~40% (Fig. 5A, T48N). These results indicated that ARF must be able to convert to its GTP-bound form in order to reposition the Golgi complex around the inclusion and that ARF-GDP has a dominant-negative effect, either directly or indirectly.

Lastly, we assessed their respective roles in MT stability. Cells overexpressing ARF1 Q71L-HA and WT ARF1 displayed normal PTM MT cages. In contrast, the overexpression of GDP-locked ARF1 T48N inhibited the formation of PTM MT cages by ~50% (Fig. 5B). Importantly, neither ARF Q71L nor ARF T48N overexpression affected the formation of alpha-tubulin cages (Fig. S8). These results indicate that, similar to Golgi complex positioning, the ability of ARF to be activated by GTP is required to regulate the formation of PTM MT cages around the inclusion. Altogether, these results suggest that repositioning of the Golgi complex and stabilization of MTs are coordinately regulated by ARF-GTP under control of CT813.

10.1128/mBio.02280-16.8 FIG S8  ARF-GDP or ARF-GTP recruitment to the inclusion does not impair alpha-tubulin cages. (Left) HeLa cells were infected with WT *Chlamydia* and transfected with WT, Q71L, or T48N ARF1-HA. Cells were fixed 18 h postinfection (hpi) and labeled with anti-HA (green) and anti-alpha-tubulin (red) antibodies. DNA was stained with Hoechst. Scale bars = 20 μm. (Right) Percentages of cells containing alpha-tubulin cages, normalized to results for alpha-tubulin cages present in ARF1 WT-HA-transfected cells from three independent experiments, ± standard deviations. A minimum of 100 cells per condition was measured for each experiment. Download FIG S8, TIF file, 0.7 MB. Copyright © 2017 Wesolowski et al. 2017 Wesolowski et al. This content is distributed under the terms of the Creative Commons Attribution 4.0 International license.

## DISCUSSION

*Chlamydia trachomatis* requires the fragmentation and repositioning of the host Golgi complex for the development of its inclusion and the generation of infectious progeny (11, 12). Because the Golgi complex is tightly regulated to ensure proper localization, transport, assembly, fragmentation, and ribbon formation (31), *Chlamydia* must work against the normal flow of Golgi complex membranes to control Golgi complex localization and dynamics during infection. ARF GTPases are major regulators of Golgi complex dynamics and structure because they control cisternal maturation, vesicular trafficking, and membrane lipid composition through the reversible association with Golgi complex membranes (25). Seminal studies using brefeldin A, which causes the collapse of the Golgi complex into the endoplasmic reticulum, illustrated the importance of ARF activation in regulating the Golgi complex structure (32–34). Thus, ARFs are ideal targets for *Chlamydia* to manipulate the Golgi complex. Here, we have demonstrated that *Chlamydia* uses the inclusion protein CT813 to hijack Golgi complex ARF GTPases ARF1 and ARF4, in order to control Golgi complex dynamics around the inclusion.

ARF GEFs activate ARF GTPases through nucleotide exchange. Importantly, GEFs also recruit ARF proteins to membranes (35). The localization of ARF GEFs to specific membranes controls when and where ARFs function. Interestingly, CT813 recruits ARF1 and ARF4 independently of other proteins (Fig. 1), and CT813 is required for *Chlamydia*-induced ARF1 activation (Fig. 4A). Although CT813 alone does not display GEF activity *in vitro* (Fig. 4B), these data show that *Chlamydia* infection induces ARF activation by a mechanism that involves its direct interaction with CT813. Considering the unusual binding properties of CT813 to ARF1, possible mechanisms could involve CT813 as a recruitment factor for a cellular GEF, or for another component that remains to be identified, which would allow CT813 to function as a GEF. In this respect, CT813 may be part of a multimeric GEF, similar to the mammalian TRAPP complex (36, 37) or the Mon1/Ccz GEF (38). The second possibility is supported by the fact that CT813 interacts with both ARF-GDP and ARF-GTP, which is a hallmark of GEFs (29), and that CT813 competes with cellular GEFs *in vitro*.

In mammalian cells, the Golgi complex is intimately associated with PTM MTs (39, 40). Golgi complex stacks disperse along PTM MTs following MT depolymerization (39). Similarly, *Chlamydia trachomatis* couples the relocation of Golgi complex ministacks around the inclusion to the PTM of inclusion-associated MTs (11). However, the molecular mechanism by which this coordination occurs has remained elusive. Our data support a model whereby *Chlamydia* utilizes the inclusion membrane protein CT813 to coordinate PTM MTs and Golgi complex repositioning around the inclusion. *Chlamydia* induces the local accumulation of PTM MTs through the CT813-dependent recruitment and activation of host ARF GTPases. It is along these PTM MTs that Golgi complex ministacks move around the inclusion.

Golgi complex fragmentation and repositioning around the inclusion is important for *Chlamydia* development by enhancing *Chlamydia*’s access to nutrients and lipids (11, 12). While the CT813 KO strain (data not shown) and the chemically mutagenized CT813 strain developed by Kokes et al. (23) do not display defects in sphingomyelin recruitment, the CT813 KO *Chlamydia* strain displays smaller inclusions, indicating a defect in inclusion development, which may involve other lipid species. Further supporting the importance of Golgi complex repositioning during infection, the CT813 KO strain also produced fewer infectious progeny. In contrast, the chemically mutagenized CT813 strain did not display a growth defect (23). This discrepancy could be due to the difference between completely knocking out a gene and mutagenesis, in which some functions may remain.

The PTM of tubulin is a consequence of MT stability (41). Interestingly, the loss of CT813, ARF1, and ARF4 profoundly affects the PTM of MT cages present around the inclusion (Fig. 2), indicating that these proteins are involved in the same pathway. To our knowledge, a direct role for ARF1 and ARF4 in the regulation of MTs has never been established. Thus, our data suggest a novel function for these isoforms in regulating MT stability. Dynamic instability is a fundamental characteristic of MTs and is regulated in part by MT-associated proteins (MAPs) (42). MAPs influence the association of other proteins, such as MT-severing proteins, with MTs (18, 43). By inhibiting MT severing, MAPs prolong the life of MTs, which can then be posttranslationally modified. Thus, CT813, ARF1, and ARF4 may play a role in the recruitment of MAPs to alpha-tubulin, which in turn acts on MT cages to influence their stability. For example, 14-3-3 proteins, which bind to acetylated tubulin (44), have also been shown to interact with CT813 (23). It will be interesting to investigate whether 14-3-3 proteins function in parallel with the CT813/ARF complex to coordinate microtubule stability. However, we cannot rule out a role for these proteins in the direct recruitment of enzymes responsible for the PTM of MTs, such as acetyltransferases and carboxypeptidases (18).

In addition to MT cages, the chlamydial inclusion is surrounded by a network of actin that maintains the integrity of the inclusion (45). CT813 has also been implicated in regulating the formation of these actin cages (Fig. S6) (23). Thus, CT813 functions as a master cytoskeletal regulator. Interestingly, MT and actin cage formation occur with distinct temporal kinetics (11, 45). How CT813 transitions from one cytoskeletal element to another is under investigation.

Examining host-pathogen interactions enabled the discovery of a new function for ARFs in regulating MT stability. It will be important to determine whether hijacking of ARF GTPases to regulate MT stability is a common pathogenic mechanism used to hijack different host cell pathways. Ultimately, identifying the molecular components involved in this pathway will shed light on how ARF1 and ARF4 regulate PTM MTs.

## MATERIALS AND METHODS

### Cell culture and transfections.

HeLa cells (ATCC) were cultured as described previously (46). Cells were transfected using Continuum transfection reagent (Gemini Bioproducts) according to the manufacturer’s instructions. For siRNA transfection, HeLa cells were transfected using 1 nM siRNA and DharmaFect I reagent (Dharmacon) according to the manufacturer’s instructions at 48 h prior to infection.

### CT813 nomenclature.

Since the function of CT813 is not limited to actin polymerization, we do not use the InaC nomenclature. CT813 is defined as CTL0184/InaC from the LVG-L2 434/Bu strain of *Chlamydia trachomatis* (nucleotides 235,152 to 235,946).

### *Chlamydia* strains.

*Chlamydia trachomatis* L2 was propagated and purified as described elsewhere (47, 48). The CT813 KO *C. trachomatis* strain was generated by retargeting the intron to CT813 via the TargeTronics algorithm and transformed as described previously (49). CT813-FLAG L2 was generated by transforming *C. trachomatis* L2 with a plasmid carrying genes encoding CT813-FLAG under control of a tetracycline promoter as described elsewhere (50). *Chlamydia* growth was assessed at 48 h as described for earlier studies (49).

### Antibodies.

The following primary antibodies were used: anti-ARF1 (mouse [mo]; Santa Cruz Biotechnology [SCBT]); anti-ARF3 (mo; SCBT); anti-actin (rabbit [rb]; Sigma); anti-acetylated alpha-tubulin (mo; Sigma); anti-alpha-tubulin (mo; Sigma); anti-HSP70 (chicken [ck]; StressMarq); anti-detyrosinated alpha-tubulin (rb; Abcam, Inc.); anti-FLAG (mo; Thermo Fisher); anti-FLAG (rb; Sigma); anti-HA (chicken; Thermo Fisher); anti-pan-ARF (mo; Millipore); anti-ARF6 (rb; Cell Signaling); anti-ARF5 (mo; Abnova); anti-ARF4 (rb; ProteinTech); anti-giantin (rb; BioLegend); anti-GM130 (mo; Becton Dickinson [BD]); anti-lipopolysaccharide (anti-LPS; mo; Virostat); anti-CT813 (rb; T. Hackstadt); anti-IncA (rb; T. Hackstadt). Goat anti-rabbit and anti-mouse IgG–Alexa Fluor488, -555, and or -647–conjugated secondary antibodies, goat anti-chicken IgY Alexa Fluor555-conjugated secondary antibody, and donkey anti-rabbit and anti-mouse IgG–horseradish peroxidase (HRP)–conjugated secondary antibodies were purchased from Invitrogen. Donkey anti-chicken IgY–HRP–conjugated secondary antibody was purchased from Pierce.

### Recombinant DNA/vector and cloning.

PCR and cloning were conducted using standard procedures and the primers listed in Table 1. GST-GGA1 was a kind gift from B. Collins (University of Queensland). FLAG-CT813 was constructed by PCR amplification of CT813 using primers FO541 and FO543 and ligation into pCMV-tag2b (a gift from P. Roche, NIH). ARF1-HA, ARF3-HA, ARF4-HA, ARF5-HA, and ARF6-HA were generated by PCR amplification from HeLa cDNA using primers FO661 to -670 and ligation into pcDNA3.1(+) containing a C-terminal HA tag. ARF1-Q71L-HA was made by PCR amplification of ARF1-Q71L (gift from J. Keen, Thomas Jefferson University) using primers FO661 and -662 and ligation into pcDNA3.1(+) containing a C-terminal HA tag. ARF1-T48N-HA was constructed using QuikChange PCR (Agilent) and the primers FO704 and -705 according to the manufacturer’s instructions. His-CT813 was constructed by PCR amplification of CT813 using primers FO397 and -398 and ligation into pET28a(+). Tetracycline-inducible CT813-FLAG for *Chlamydia* transformation was constructed by PCR amplification of CT813 with primers FO498 and -499 and ligation into pBOMB4-Tet (46, 50).

**TABLE 1:** List of primers

Primer	Sequence (5′–3′)	Construct
FO397	GGGCATATCCATATGACTACTCTTCCCAATAC	6×His-CT813
FO398	ACGCGTCGACTCACTATATCGAACCACGTCTTCC	

FO498	AAGGAAAAAAGCGGCCGCATGACTACTCTTCCCAATAATTG	pBomb4-CT813-Tet
FO499	ACGCGTCGACCTACTTGTCATCGTCATCCTTGTAGTCTATCGAACCACGTCTTCC	

FO541	AACTGCAGATGACTACTCTTCCCAATACTTGTACTTCA	Flag-CT813
FO543	CCCAAGCTTCTATATCGAACCACGTCTTCCTGG	

FO661	CGAGGTACCATGGGGAACATCTTCGCC	ARF1 WT-HA
FO662	CTGCAACTCGAGCTTCTGGTTCCGGAGCTGATT	ARF1 Q71L-HA
FO663	CGAGGTACCATGGGCAATATCTTTGGAAACC	ARF3-HA
FO664	CTGCAACTCGAGCTTCTTGTTTTTGAGCTGATTGGC	

FO665	CTAGGTACCATGGGCCTCACTATCTCCTCC	ARF4-HA
FO666	CTGCAACTCGAGACGTTTTGAAAGCTCATTTGACAG	

FO667	ATAGGTACCATGGGCCTCACCGTGTCC	ARF5-HA
FO668	CTGCAACTCGAGGCGCTTTGACAGCTCGTG	

FO669	ATAGGTACCATGGGGAAGGTGCTATCCAAAA	ARF6-HA
FO670	CTGCAACTCGAGAGATTTGTAGTTAGAGGTTAACCATGTG	

FO704	GATCGTGACCACCATTCCCAACATAGGCTTCAACGTGGAAACCGTGG	ARF1 T48N-HA
FO705	GTTTCCACGTTGAAGCCTATGTTGGGAATGGTGGTCACGATCTCACCC	

FO708	CGTCTCCTTTGAGCTGTTTGC	hPPIA RT-PCR
FO709	TTGACACTTCCTGGGACTGG	

FO710	CGTGTTTGCTGTGAAGACGGT	hARF1 RT-PCR
FO711	ACGCTCTCTGTCATTGCTGT	

FO712	GAGGGAGCGGAGCGGAAC	hARF3 RT-PCR
FO713	GCATTAGGCAGATCCTGTTTGTTTG	

FO714	TGCTTCTGCCCATCACAAGT	hARF4 RT-PCR
FO715	AGCATCCAATCCAACCATCA	

FO716	ATGCGGATTCTCATGGTTGG	hARF5 RT-PCR
FO717	TCAGCAGATTCTTGGACCCG	

### siRNA.

SmartPool ON-TARGETplus human ARF1, ARF4, and nontargeting control siRNA were purchased from Dharmacon. The siRNA sequences are shown in Table 2.

**TABLE 2:** List of siRNAs

siRNA target	siRNA sequence (5′–3′)	Catalog no.
Nontargeting control	UGGUUUACAUGUCGACUAA	D-001810-01-05

hARF1 SMARTpool	UGACAGAGAGCGUGUGAAC	J-011580-05
	CGGCCGAGAUCACAGACAA	J-011580-06
	ACGAUCCUCUACAAGCUUA	J-011580-07
	GAACCAGAAGUGAACGCGA	J-011580-08

hARF4 SMARTpool	AGACAACCAUUCUGUAUAA	J-011582-05
	GCUAUGGCCAUCAGUGAAA	J-011582-06
	GAACUGGUCUGUAUGAAGG	J-011582-07
	GGGCUUCAGUCUCUUCGUA	J-011582-08

### RT-PCR.

RNA was extracted using the RNeasy kit (Qiagen). Reverse transcription-PCR (RT-PCR) was performed using the Verso 1-step RT-PCR kit (Thermo Fisher) with 100 ng of RNA and 200 nM each primer (FO708 to -717). Primer sequences are listed in Table 1.

### Immunoprecipitation.

For the immunoprecipitation experiments, HeLa cells were lysed in cold lysis buffer (50 mM Tris, 100 mM NaCl, 2 mM MgCl_2_, 1% NP-40, 10% glycerol [pH 7.5] supplemented with 2 mM phenylmethylsulfonyl fluoride, 2 μg/ml pepstatin A, 1 μg/ml leupeptin, 50 mM NaF, and 1 mM Na_3_VO_4_) for 1 h on ice. Lysates were clarified by centrifugation, and equal amounts of total protein were incubated overnight at 4°C with anti-FLAG antibody immobilized on protein G Plus agarose beads. Beads were washed with lysis buffer, boiled in Laemmli buffer, and analyzed by Western blotting.

### Western blotting.

Samples for our Western blotting analysis were separated on 10% or 4-to-12% bis-Tris SDS-PAGE gels (Invitrogen) and transferred to polyvinylidene difluoride membranes for 1 h at 100 V and 4°C. Blotting was performed as described elsewhere (51). To quantify changes in tubulin levels during infection, the ratio of tubulin to HSP70 was obtained for each noninfected and infected sample. The data were then normalized to the results with the noninfected sample, which were set as 100%.

### Immunofluorescence.

For our immunofluorescence analysis, HeLa cells were fixed with 4% paraformaldehyde in cytoskeleton buffer (10 mM morpholineethanesulfonic acid, 138 mM KCl, 3 mM MgCl_2_, 2 mM EGTA, 0.32 M sucrose; pH 6.1) for 20 min. All incubations were performed at room temperature. Cells were permeabilized with either (i) 0.05% saponin in blocking buffer (10% goat serum, 0.1% bovine serum albumin in phosphate-buffered saline [PBS; pH 7.4]) for 1 h for experiments with anti-HA, anti-FLAG, anti-giantin, anti-GM130, anti-IncA, and anti-LPS antibodies or (ii) with 0.5% Triton X-100 in PBS for 10 min followed by three washes with 0.1% Triton X-100 in PBS and incubated for 1 h in blocking buffer for experiments with anti-acetylated tubulin, anti-detyrosinated tubulin, and anti-alpha-tubulin antibodies. Coverslips were incubated with primary antibodies diluted in blocking buffer containing the appropriate detergent for 1 h. Following several washes, coverslips were incubated with Alexa Fluor-conjugated secondary antibodies and Hoechst stain for 1 h. Coverslips were then washed and mounted with ProLong Diamond antifade reagent (Invitrogen). Images were acquired using a Nikon TiE inverted fluorescence microscope with a 60× oil immersion lens and Elements software (Nikon). Images were processed using ImageJ (NIH). To measure the length of the Golgi complex, *z*-stacks were acquired in 0.3-μm sections and deconvolved. Length measurements were acquired by tracing the Golgi complex of infected cells from maximum intensity projections to capture the Golgi complex in all planes via the Elements software (Nikon). A minimum of 100 cells per condition for each experiment was measured.

### ARF1-GTP binding assay.

For the ARF1-GTP binding assay, HeLa cells were infected for 24 h with WT or CT813 KO *Chlamydia* strains. The cells were then lysed as described above. Lysates were clarified by centrifugation, and equal amounts of total protein were incubated with recombinant GST-GGA1-GAT immobilized on glutathione-agarose beads for 1 h at 4°C. Following several washes, the beads were boiled in Laemmli buffer and samples were analyzed by Western blotting.

### Recombinant protein purification.

Human ^myr^ARF1 was coexpressed in *Escherichia coli* cells with yeast *N*-myristoyl transferase (NMT) and purified as described previously (52). N-terminal His-tagged full-length human ARNO (3G isoform) and ARNO^Sec7^ constructs were overexpressed in *Escherichia coli* and purified on a Ni-nitrilotriacetic acid (Ni-NTA) affinity column, followed by size exclusion chromatography as described previously for ARNO^Sec7^ (53). The 6×His-TEV-human Brag2^Sec7PH^ protein (residues 390 to 763) (54) was purified by Ni-NTA affinity chromatography and gel filtration.

### Liposome reconstitution.

Lipids were obtained from Avanti Polar Lipids. Liposomes were prepared for reconstitution as described previously in a buffer containing 50 mM HEPES, 200 mM KCl, 1 mM dithiothreitol (DTT), 10% glycerol, pH 7.4 (55). Liposomes contained 38% phosphatidylcholine, 20% phosphatidylethanolamine, 20% phosphatidylserine, 2% phosphatidylinositol-3,4,5-triphosphate, and 20% cholesterol and were extruded through a 0.2-μm filter (Whatman). Detergent-purified CT813 was incorporated into liposomes by dialysis, and the protein-containing liposomes were purified by flotation on a sucrose gradient. All flotation (centrifugation for 1 h at 55,000 rpm) and cosedimentation (saccharose-containing liposomes centrifuged for 30 min at 100,000 rpm) experiments were performed in 50 mM HEPES, 200 mM KCl, 1 mM MgCl_2_, 1 mM DTT, 10% glycerol, pH 7.4.

### Kinetic measurements of nucleotide exchange.

Activation of ^myr^ARF1 was monitored by FRET between the tryptophans of ARF1 and mantGTP (emission and excitation wavelengths of 292 and 440 nm, respectively) at 37°C in 50 mM HEPES, 200 mM KCl, 1 mM MgCl_2_, 1 mM DTT, 10% glycerol, pH 7.4. Empty liposomes or CT813-liposomes (100 μM) were incubated for 2 min at 37°C with 0.4 μM ^myr^ARF1 and 5 nM Brag2 or 20 nM ARNO, before the addition of 5 μM mantGTP. The reverse reaction was monitored by addition of 100 μM GDP to mantGTP-loaded ARF1. Activation of Δ17ARF1 by ARNO^Sec7 ^was monitored under the same conditions with 1 µM Δ17ARF1 and 50 nM ARNO^Sec7^.
