# Supplementary figures and images for: Chlamydia Hijacks ARF GTPases To Coordinate Microtubule Posttranslational Modifications and Golgi Complex Positioning
Source: mBio. 2017 May 2;8(3):e02280-16. doi: 10.1128/mBio.02280-16 (PMC5414008; doi:10.1128/mBio.02280-16)

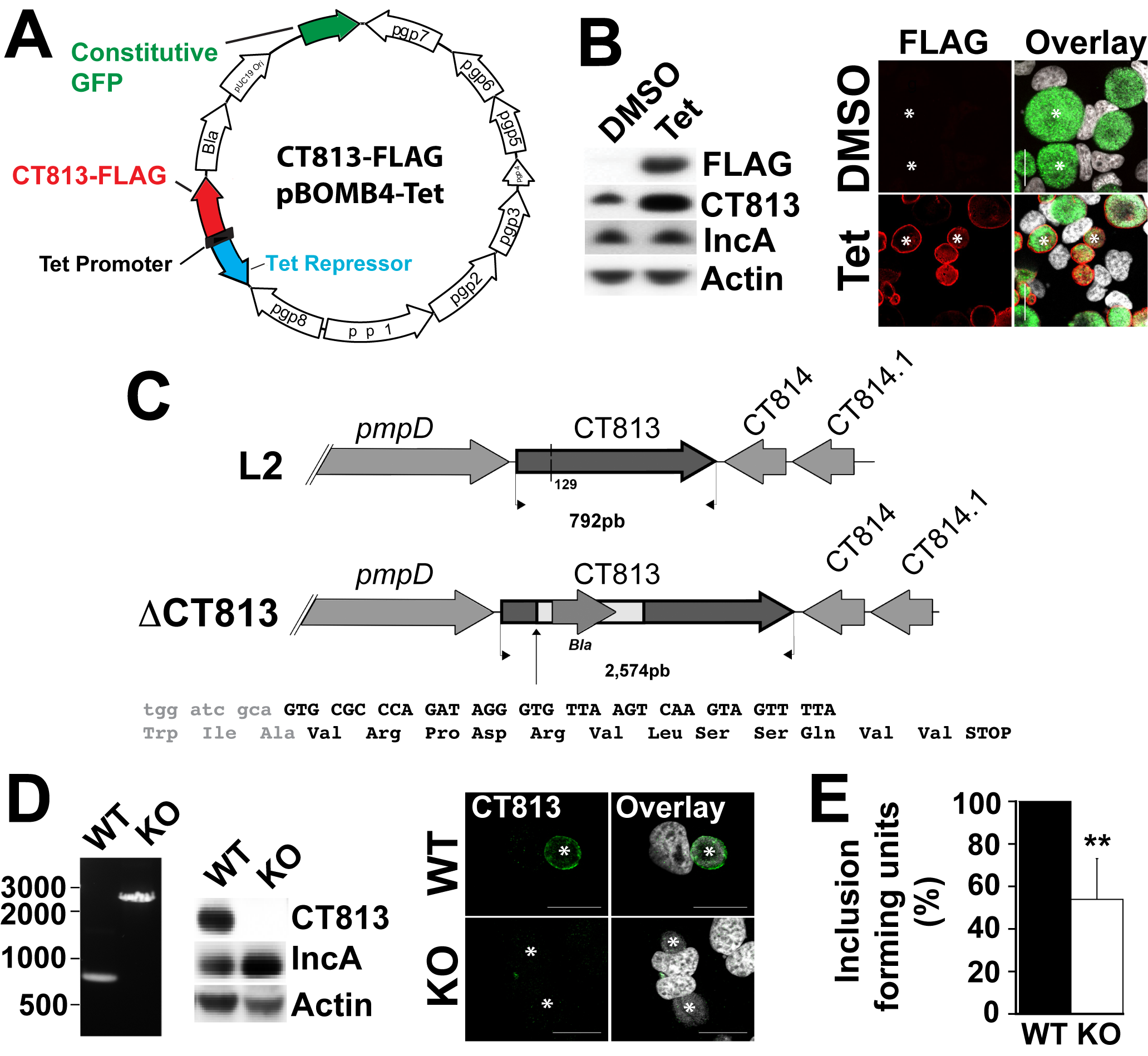

Supplement: FIG S1 [file mbo002173285sf1.tif]

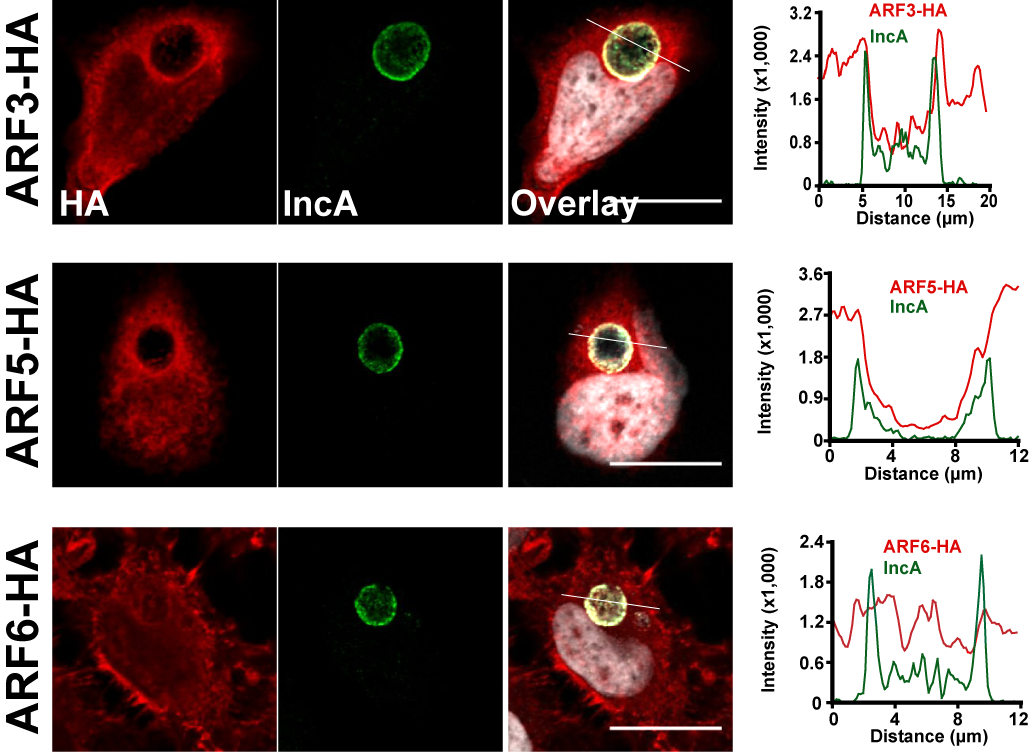

Supplement: FIG S2 [file mbo002173285sf2.tif]

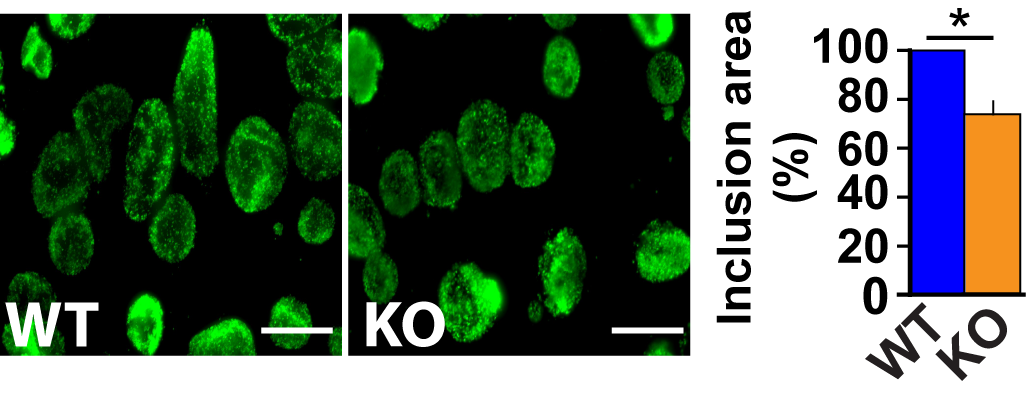

Supplement: FIG S3 [file mbo002173285sf3.tif]

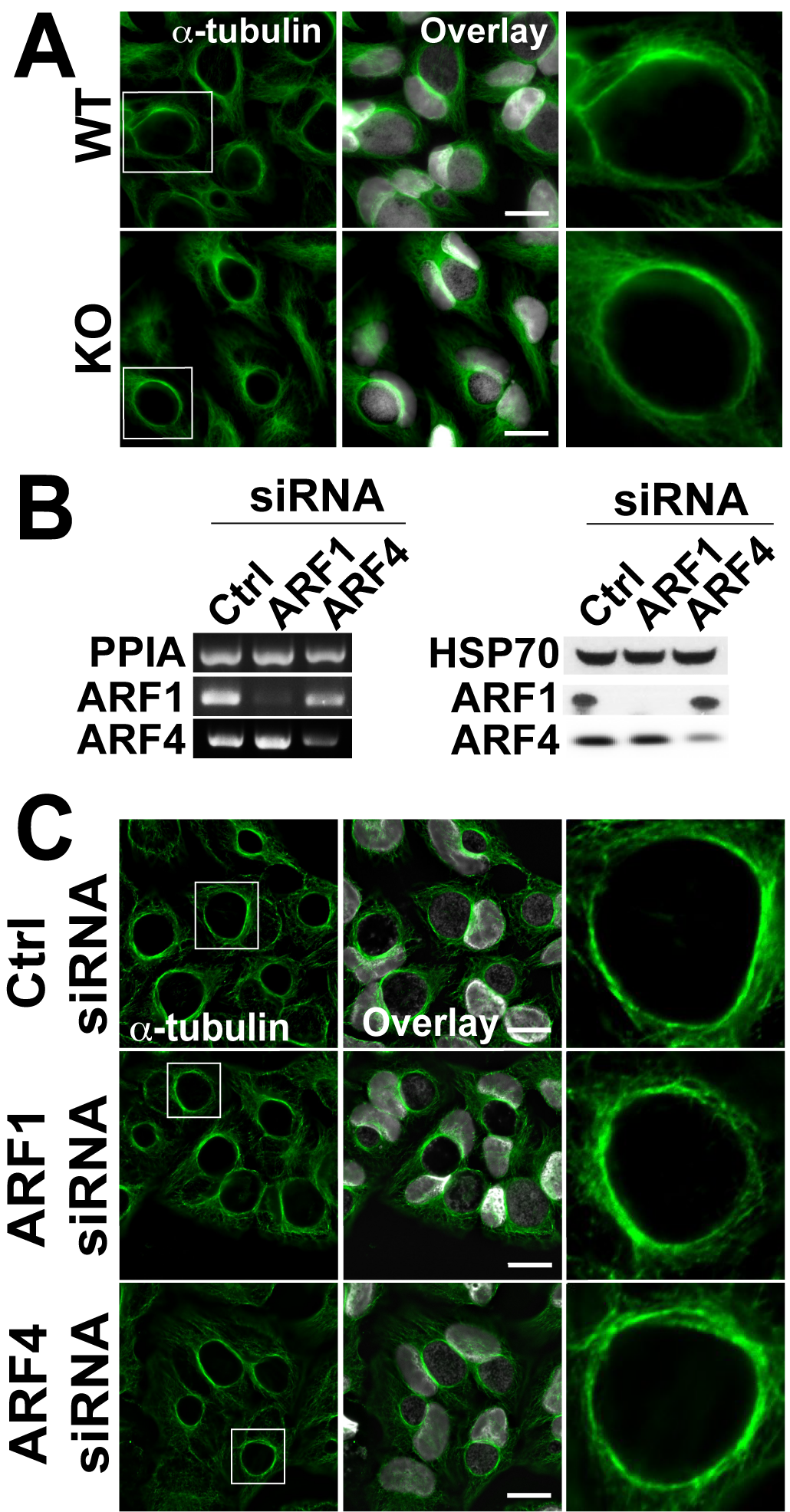

Supplement: FIG S4 [file mbo002173285sf4.tif]

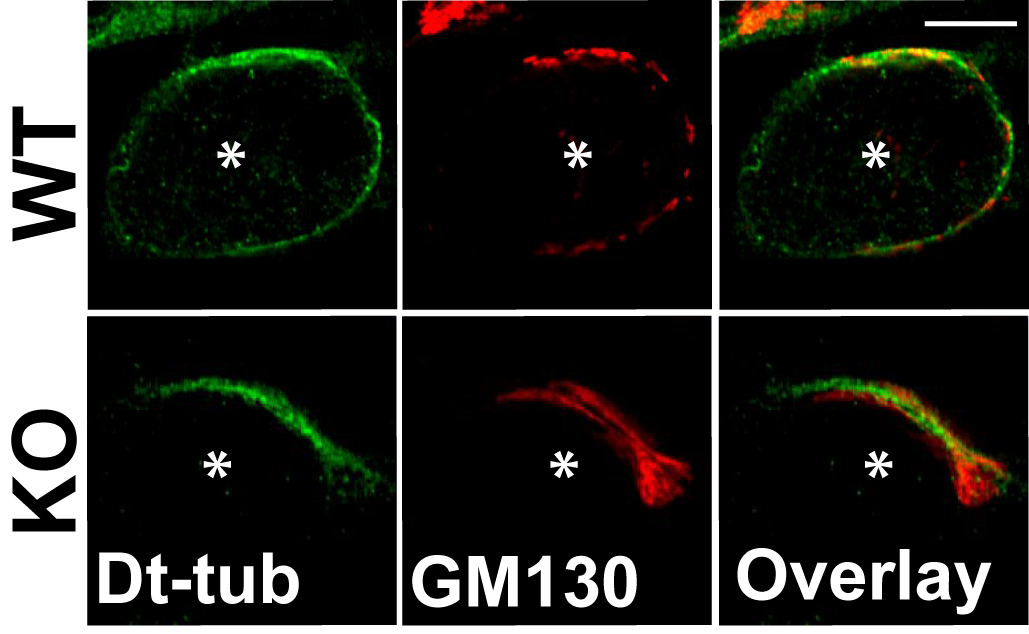

Supplement: FIG S5 [file mbo002173285sf5.tif]

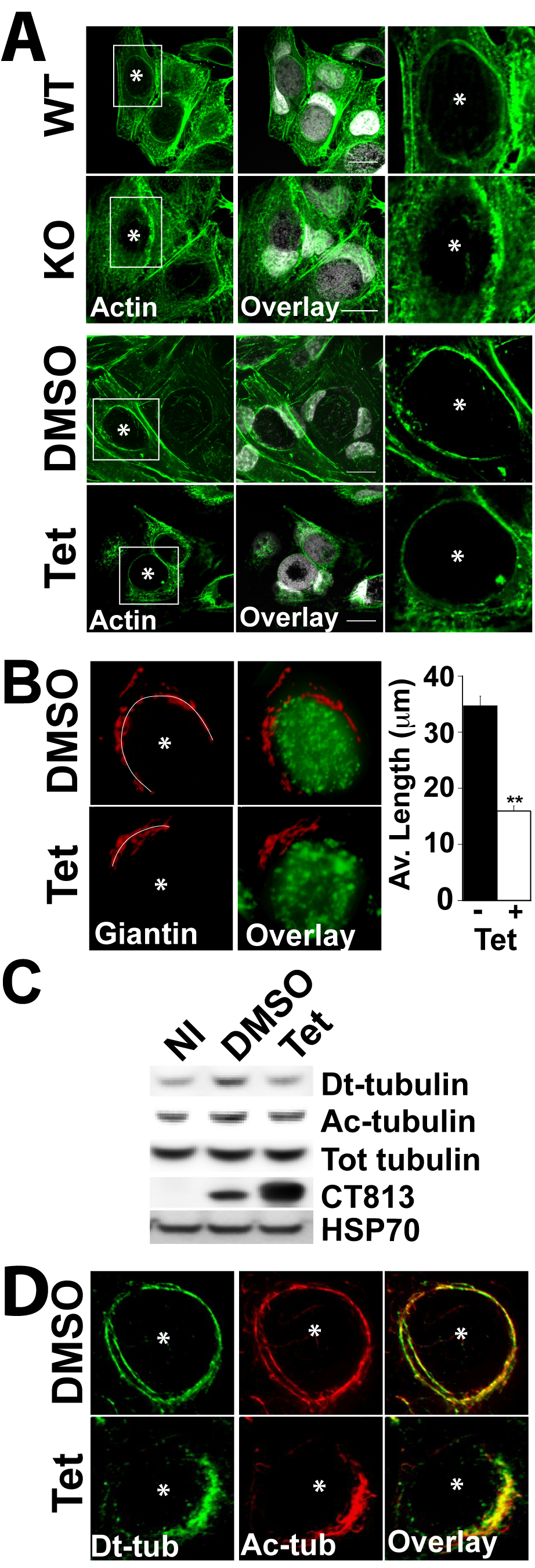

Supplement: FIG S6 [file mbo002173285sf6.tif]

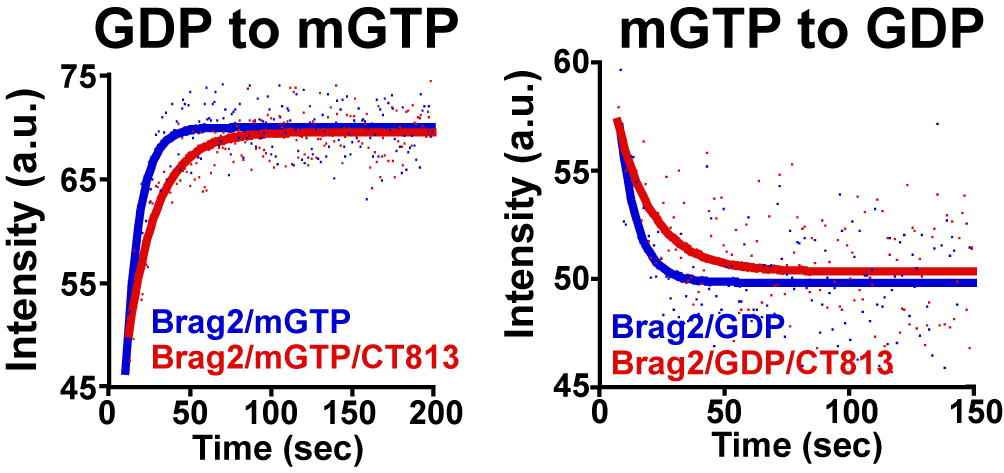

Supplement: FIG S7 [file mbo002173285sf7.tif]

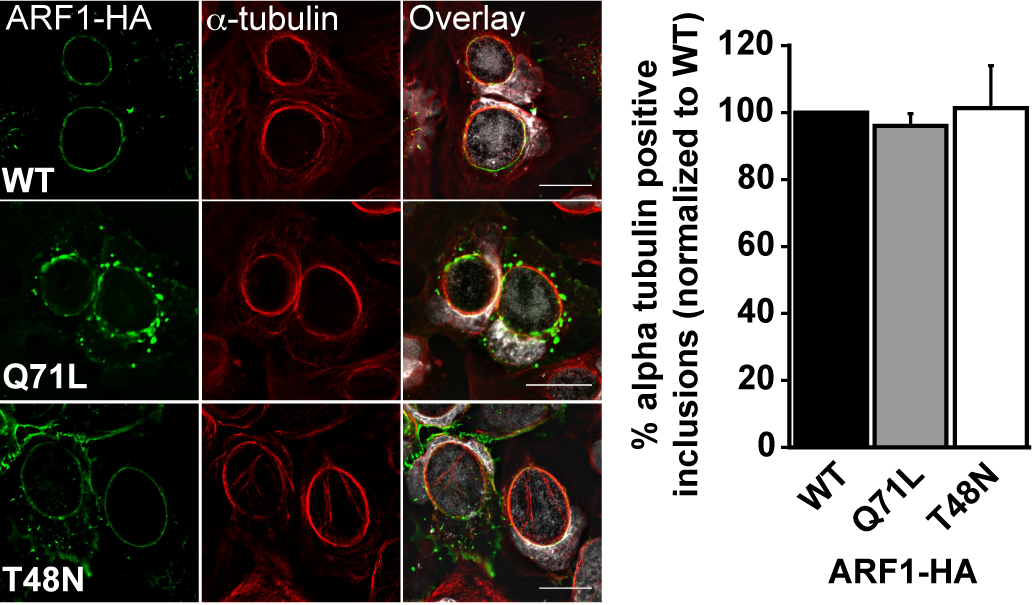

Supplement: FIG S8 [file mbo002173285sf8.tif]
